# Viral Infection and Apoptosis

**DOI:** 10.3390/v9120356

**Published:** 2017-11-23

**Authors:** Marc Kvansakul

**Affiliations:** Department of Biochemistry and Genetics, La Trobe Institute for Molecular Science, La Trobe University, Melbourne, VIC 3086, Australia; m.kvansakul@latrobe.edu.au; Tel.: +61-3-94792263

Viruses are master molecular manipulators, and evolved to thrive and survive in all species. Key to their continuing success has been the ability to subvert host cell defence systems to ensure viral survival, replication and proliferation. Amongst the diverse arsenal of defence mechanisms deployed by multicellular hosts are those that rely on rapid activation of programmed cell death or apoptosis pathways, to trigger premature cell death of infected host cells. Apoptosis of an infected host cell has been identified as a powerful mechanism to curtail viral spread, and consequently, viruses have evolved sophisticated molecular strategies to subvert host cell apoptotic defences.

This special issue is devoted to the interplay of viruses and host cell apoptotic signalling pathways. Many viruses manipulate apoptosis for their own purpose, and Zhou et al. review the interplay of viruses with death receptor mediated apoptosis [1]. This contribution is complemented by a companion review focussed on the role of the intrinsic or Bcl-2 mediated pathway of apoptosis in host–virus interactions by Kvansakul et al. [2], as well as by a research article examining the structure and function of a canarypoxvirus encoded Bcl-2 homolog from Anasir et al. [3]. The ability of other poxviruses to modulate apoptosis signalling is discussed by Nichols et al. [4]. From the large group of DNA viruses that have been shown to subvert apoptosis signalling, Epstein–Barr virus is examined by Fitzsimmons & Kelly [5], with another prominent herpesvirus, cytomegalovirus, being reviewed by Brune & Andoniou [6]. This set of reviews is completed by an overview from Dixon and colleagues on the African swine fever virus-encoded modulators of apoptosis [7]. Influenza virus interference with apoptosis is reviewed by Shim et al. [8], which is complemented by a research article from Othumpangat et al. [9] describing the role of miRNA-4776 in regulating host cell NF-κB signalling. Moving onto RNA viruses, the interplay between flaviviruses and cell death pathways is reviewed by Okamoto et al. [10], whereas HIV induced bystander apoptosis is reviewed by Garg & Joshi [11]. Another important RNA virus, hepatitis B virus, is reviewed by Lin & Zhang [12], with a final research article describing the establishment of an Avian infectious bronchitis cell culture system by Han et al. [13].

I hope that the research articles and reviews that were assembled for this special issue will shed light for both experts and interested bystanders on the complex and sometimes unexpected interplay between viruses and host cell apoptosis signalling pathways, and inspires researchers to investigate the many unresolved questions about the ways and means utilized by viruses to manipulate life and death of an infected cell.
